# Fear of mammography and correlated psychosocial factors

**DOI:** 10.1192/j.eurpsy.2026.11543

**Published:** 2026-07-31

**Authors:** M. Theodoratou, K. Paraskeva, V. Yotsidi, E. Ferentinou, P. Andriopoulou, N. Nikitidis, G. Tsitsas, E. Toki, S. Papadopoulou, C. Dafogianni

**Affiliations:** 1 Social Sciences; 2 Hellenic Open University, Patras; 3 Psychology, Panteion University; 4 Nursing, University of West Attica, Athens; 5 Psychology, University of Ioannina, Ioannina; 6 Charokopeion University, Athens; 7 University of Ioannina, Ioannina, Greece

## Abstract

**Introduction:**

Psychosocial factors influence women’s fear and attitudes toward mammography. Understanding these factors is crucial for increasing participation in early detection and reducing breast cancer mortality. The research focuses on psychological mechanisms, such as fear of a cancer diagnosis, perceived personal risk, and self-efficacy, as well as social influences like support networks and cultural beliefs. Practical barriers, including pain during the procedure and limited access to screening facilities, are also examined.

**Objectives:**

This study investigates the psychosocial factors influencing women’s fear and attitudes toward mammography, aiming to understand why many women hesitate or avoid preventive screening.

**Methods:**

Data were collected from 274 women using three questionnaires: a socio-demographic survey; the Champion Health Belief Model Scale (CHBMS, 1984/1999), assessing perceived susceptibility, severity, benefits, barriers, self-efficacy, health motivation, and cues to action regarding mammography; and the Mammography Questionnaire (MGQ), evaluating user satisfaction with the screening process.

**Results:**

The sample consisted mainly of married, middle-aged women (35–54 years) with relatively high education, many employed in the public sector, living in urban areas, and generally positive about health and psychological well-being. Overall, 73.7% reported few or no difficulties in accessing screening. Women demonstrated high awareness of early detection, strong motivation to protect their health, and confidence in mammography’s effectiveness. Notably, older women exhibited pronounced fear of the procedure. Physician recommendation emerged as the most influential factor prompting women to undergo mammography.

**Conclusions:**

Women are highly aware of the importance of early breast cancer detection and motivated to participate in preventive screening. Addressing fear, particularly among older women, and leveraging healthcare professionals’ guidance are critical for improving participation rates and early diagnosis

**Disclosure of Interest:**

None Declared

